# Adult-Onset Immunoglobulin A Vasculitis With Renal Involvement

**DOI:** 10.7759/cureus.23649

**Published:** 2022-03-30

**Authors:** Si Yuan Khor, Abdul-Fatawu Osman, Issa Haddad, Sara AlAttal, Nazia Khan

**Affiliations:** 1 Internal Medicine, Michigan State University, Lansing, USA

**Keywords:** atypical rash, iga nephritis, henoch-schönlein purpura (iga vasculitis), adult iga vasculitis, lekocytoclastic vasculitis

## Abstract

A 50-year-old male presented with worsening bilateral lower extremities swelling for a month, associated with a purpuric rash over bilateral upper and lower extremities, joint pain over bilateral hands and ankles, and intermittent generalized abdominal pain. Physical examination was notable for pitting edema in bilateral lower extremities and palpable, non-blanching purpuric rashes and crusts, joint tenderness over bilateral hands/wrists/ankles, and mild generalized abdominal tenderness. Laboratory tests were remarkable for sub-nephrotic range proteinuria and microscopic hematuria. The skin biopsy revealed leukocytoclastic vasculitis. Renal biopsy showed mild mesangial expansion and immunoglobulin A (IgA)-dominant mesangial deposits. The patient was diagnosed with IgA vasculitis (IgAV) nephritis (IgAVN) and was subsequently treated with oral prednisone 80 mg daily for seven days followed by slow tapering doses, oral lisinopril 2.5 mg daily, and oral furosemide 40 mg daily. At the one-month follow-up as an outpatient, his skin rash and lower extremity swelling had resolved along with an improvement of proteinuria.

## Introduction

Immunoglobulin A vasculitis (IgAV) is the most common systemic vasculitis in children but is extremely rare in adults, with an incidence rate of 0.1-1.8 per 100,000 adults [1]. Unlike in children, adult-onset IgAV is associated with a more severe clinical course, higher relapse rate, and poorer renal outcomes [2]. Misdiagnosis and failure to intervene with timely management and treatment would result in devastating long-term comorbidities and complications. In this report, we present a case of adult-onset IgAV with renal involvement and briefly discuss the currently available literature on this uncommon disease.

## Case presentation

A 50-year-old male with a past medical history of chronic tobacco use, hypertension, chronic obstructive pulmonary disease, and alcoholic cardiomyopathy presented to the emergency department with worsening bilateral lower extremities swelling for a month. He had initially developed a painless, non-pruritic, purpuric rash over bilateral upper and lower extremities three months before presentation. It had been associated with joint pain over bilateral hands and ankles without any obvious swelling or deformity. Subsequently, this had been followed by progressive worsening of bilateral leg swelling and intermittent abdominal pain. The patient denied any alopecia, oral ulcers, shortness of breath, chest pain, easy bruising/bleeding, hematuria, bloody bowel movement, altered bowel movement, photosensitivity, insect bites, appetite change, fever, weight loss, and recent infection or medication changes. He had no history of renal disease and denied any chronic non-steroidal anti-inflammatory drugs (NSAIDs) use. Also, he had no significant family history of renal disease or autoimmune disease. The patient had stopped drinking alcohol more than five years ago.

**Figure 1:**
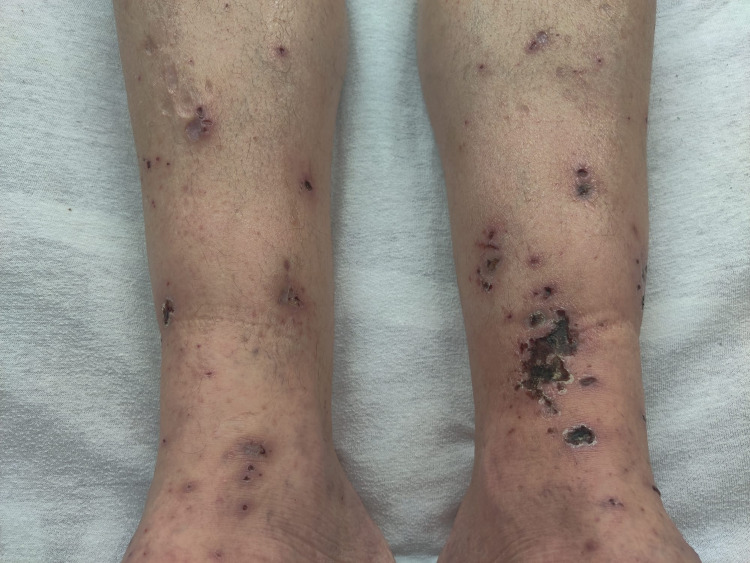
Palpable purpuric rashes and crusts over bilateral lower extremities

Upon presentation, the patient's blood pressure was 140/84 mmHg, heart rate was 80 beats per minute, respiratory rate was 18 breaths per minute with SpO_2_ of 97% on room air, and his temperature was 37 °C. Physical examination was remarkable for bilateral lower extremities pitting edema and palpable, non-blanching purpuric rashes and crusts (Figure 1), joint tenderness over bilateral hands/wrists/ankles, and mild generalized abdominal tenderness without guarding or rigidity. Otherwise, the patient did not exhibit any signs of chronic liver disease on physical examination.

Investigations

Initial laboratory studies were remarkable for hypoalbuminemia (serum albumin of 3.1 g/dL) and elevated serum erythrocyte sedimentation rate (ESR) (30 mm/hour). Urinalysis revealed proteinuria >500 mg/dL and microscopic hematuria. However, his complete blood count (CBC), comprehensive metabolic panel (CMP), coagulation profile, brain natriuretic peptide (BNP), and C-reactive protein (CRP) were unremarkable. Initial blood work on presentation showed white blood count of 9.4 x 10^3^/uL, hemoglobin of 13.4 g/dL, platelet count of 252 x 10^3^/uL, blood urea nitrogen of 7 mg/dL, creatinine of 1.0 mg/dL (within patient's baseline), sodium of 139 mEq/L, potassium of 4.9 mEq/L, chloride of 108 mEq/L, aspartate aminotransferase (AST) of 14 U/L, alanine aminotransferase (ALT) of 10 U/L, total bilirubin of 0.3 mg/dL, international normalized ratio (INR) of 1.0, prothrombin time (PT) of 11.3 seconds, activated partial thromboplastin time (aPTT) of 25.6 seconds, BNP of 40 pg/mL, and CRP of <1.0 mg/dL. X-rays of bilateral hands revealed no abnormalities. Venous Doppler ultrasound of bilateral lower extremities showed no evidence of deep vein thrombosis; 24-hour urine protein showed sub-nephrotic-range proteinuria: 2.7 g/day.

**Figure 2:**
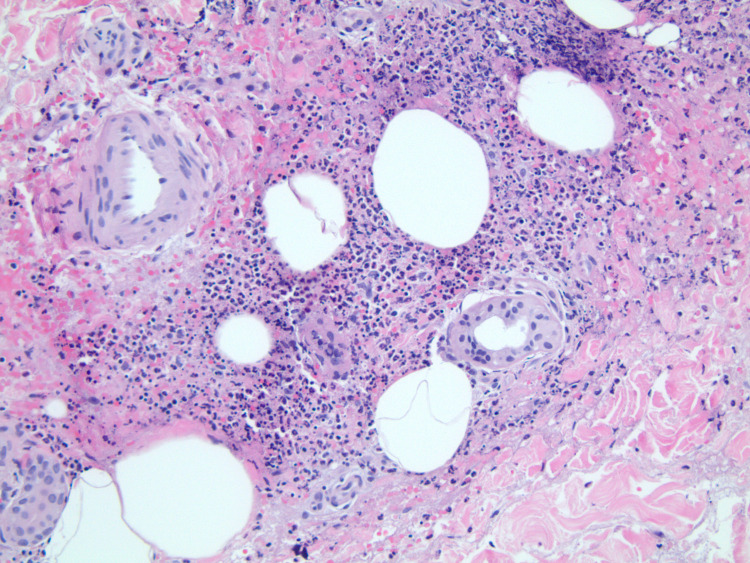
Skin biopsy showed small vessel vasculitis with surrounding nuclear dust and predominant neutrophilic infiltrate of urticarial leukocytoclastic vasculitis

**Figure 3:**
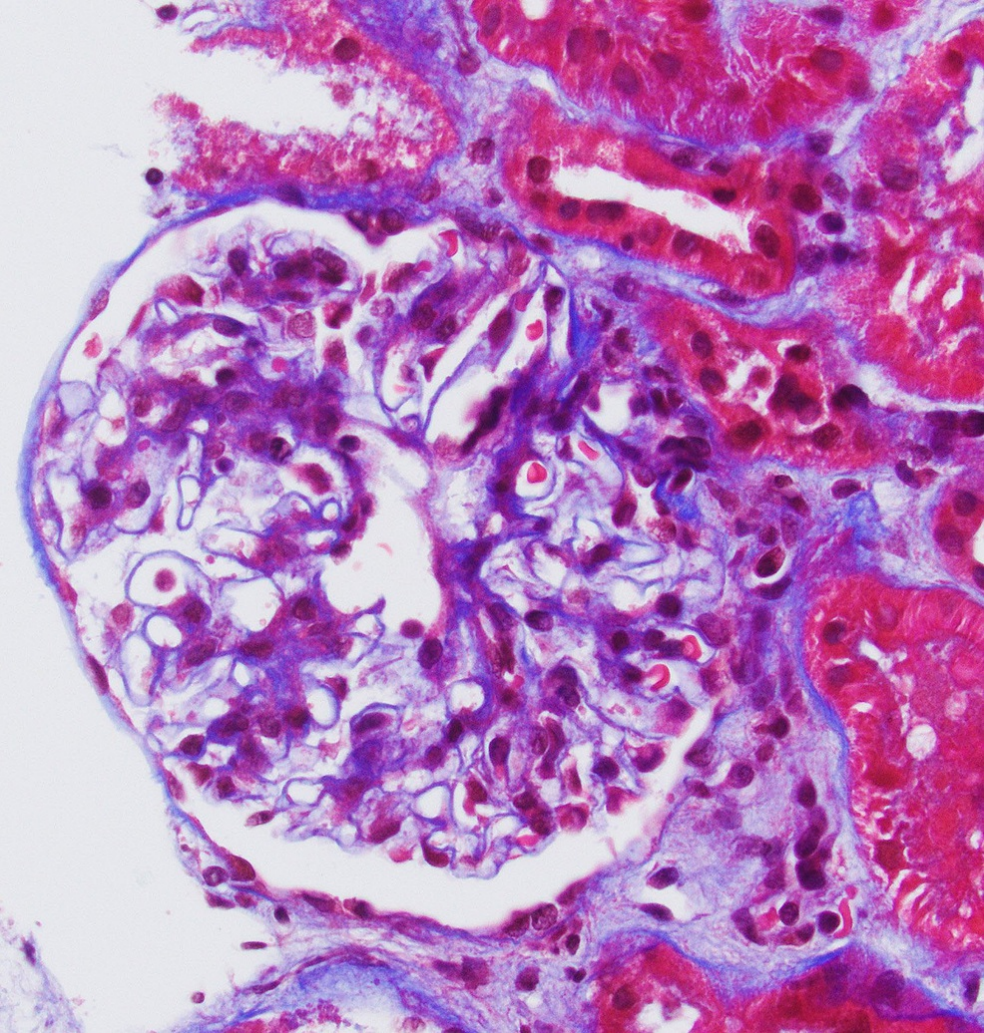
Renal biopsy showed trichome-stained glomerulus with mild mesangial hypercellularity

**Figure 4:**
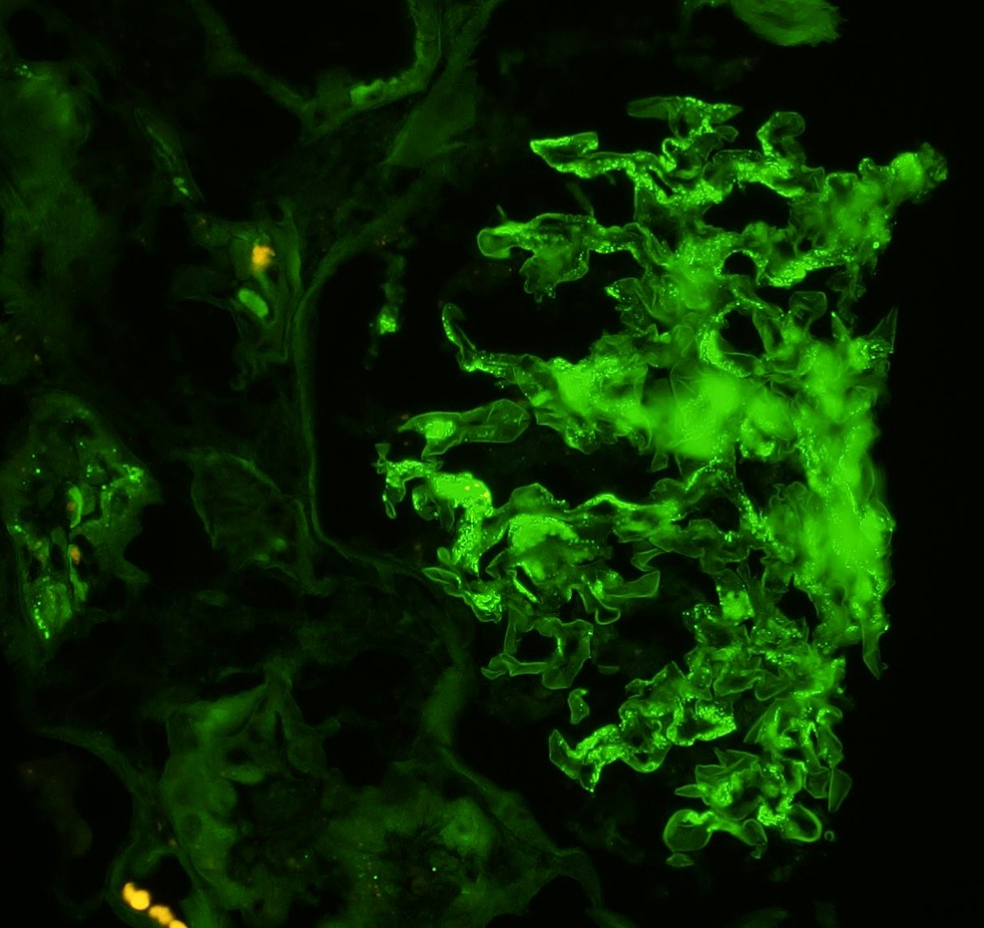
Immunofluorescence examination of renal biopsy demonstrated granular mesangial staining with IgA IgA: immunoglobulin A

Further workups, including serum antinuclear antibody (ANA), anti-double-stranded DNA antibody (Anti-dsDNA), rheumatoid factor, antineutrophil cytoplasmic antibody (ANCA), C4 and C4 complement, anti-glomerular basement membrane antibody (anti-GBM), serum IgA level, cryoglobulin, hepatitis B and C, HIV, and serum protein electrophoresis, were unremarkable. Skin biopsy of lower extremity purpuric rash was performed and it revealed small vessel vasculitis with surrounding nuclear dust and predominant neutrophilic infiltrate of urticarial leukocytoclastic vasculitis (Figure 2). Subsequent renal biopsy revealed mild mesangial expansion and proliferation on light microscopy (Figure 3), and IgA-dominant mesangial deposits by immunofluorescence (Figure 4).

Differential diagnosis

The differential diagnosis included systemic lupus erythematosus (SLE), ANCA-associated vasculitis, hypersensitivity vasculitis, cryoglobulinemic vasculitis, and immune thrombocytopenic purpura (ITP). SLE was deemed unlikely given negative serum ANA and anti-dsDNA. ANCA-associated vasculitis was also unlikely as serum c-ANCA and p-ANCA were unremarkable and biopsy results did not show necrotizing granulomas. Normal platelet counts on presentation essentially excluded ITP as a differential diagnosis. Hypersensitivity vasculitis was very unlikely given no recent change in medications and negative infectious status for HIV and hepatitis C. Cryoglobulinemic vasculitis was also ruled out due to negative hepatitis C status and negative serum cryoglobulin.

Treatment and outcome

The patient was treated with oral lisinopril 2.5 mg daily for proteinuria and oral furosemide 40 mg daily to help alleviate lower extremities edema. He was also started on oral prednisone 80 mg daily for seven days followed by slow tapering: 60 mg daily for eight weeks, 50 mg daily for four weeks, with a plan to further decrease dosage by 10 mg daily every four weeks. At the one-week follow-up as an outpatient, the patient reported that his symptoms, which included skin rash, arthralgia, abdominal pain, and lower extremities swellings, had greatly improved. At the one-month follow-up, his skin rash had completely resolved. Blood pressure was well-controlled and lower extremities edema had resolved. Improvement of proteinuria was also noted with urine protein/creatinine ratio (UPCR) at 1.7 g/day. At the three-month follow-up, UPCR was 0.6 g/day, indicating a further improvement in his proteinuria. Unfortunately, the patient was unable to follow up regularly with his nephrologist and primary care thereafter due to multiple hospital admissions for his underlying comorbid medical conditions.

## Discussion

IgAV, previously known as Henoch-Schonlein purpura is a systemic immune complex-mediated disease that mainly affects the small vessels, with characteristic IgA1-dominant immune complex deposits [3]. IgAV is predominantly a childhood disease and is the most common systemic vasculitis in children, with an annual incidence of 3-26 per 100,000 children, and the mean age of onset is six years [1]. On the contrary, IgAV is rarely seen in adults, with an annual incidence of 0.1-1.8 per 100,000 adults, and the median age of onset is 50 years [1].

The exact etiology of IgAV remains unknown and is believed to be multifactorial with immunogenicity, genetic, and environmental factors all seemingly playing a role. Several antigenic stimuli have been described to trigger this pathological process. These include infection, medications, vaccinations, certain autoimmune diseases, and tumor antigens associated with malignancies. Up to 90% of cases were reportedly preceded by viral or bacterial infections one to three weeks prior to the onset of vasculitis, most commonly an upper respiratory tract infection caused by group A Streptococcus [1]. Malignancies associated with IgAV are primarily solid tumors (61%), with lung cancer being the most common associated cancer (25%), followed by prostate (16%), renal (6%), and hematological malignancies [3]. The etiology was unclear in our patient as he neither had any prior infection nor any recent exposure to new medications.

IgAV is a form of leukocytoclastic vasculitis that predominantly affects the small vessels. The proposed pathophysiology of IgAV involves a trigger from exposure to certain allergens or antigens that results in the stimulation of IgA production. This in turn leads to the deposition of IgA immune complexes, predominantly IgA1 in the small vessels, which activate the alternate complement pathways, leading to polymorphonuclear leukocytes accumulation, ultimately resulting in vascular inflammation and damage.

The classic clinical manifestations of IgAV include the tetrad of non-thrombocytopenic palpable purpura, arthralgia/arthritis, abdominal pain, and renal involvement, which was present in our patient. These manifestations may develop over days or weeks and may vary in the order of presentation and onset [3]. A retrospective study of 250 adult patients with IgAV by Pillebout et al. reported that palpable purpuric rash (96%) was the most common manifestation on presentation. This was followed by joint involvement (61%), gastrointestinal involvement (48%), and renal impairment (32%) [2]. Besides the typical cutaneous palpable purpura, necrotic or hemorrhagic purpura, bullous and pustules have been described, especially in adults [2]. In contrast with children, in whom purpuric rashes typically involve lower extremities, upper extremities and the trunk are frequently involved in adults [2]. Joint involvements are usually transient, oligoarticular, and non-destructive. They typically involve the large joints of the lower extremities, but small joints of the upper extremities have also been reported, although less commonly [2].

Gastrointestinal manifestations classically present as colicky abdominal pain, nausea, vomiting, or even bloody stools and melena in severe cases. These are due to submucosal hemorrhage, bowel edema, and bowel ischemia. Associated severe complications include gastrointestinal bleeding, bowel ischemia, and perforation. Intussusception, which is commonly reported in children, is rarely seen in adults. Renal involvements are more common in adults and tend to be more severe and associated with poorer outcomes, affecting 45-85% of cases [2]. Presentations range from microscopic hematuria and proteinuria of varying severity to nephrotic range proteinuria and macroscopic hematuria.

IgAV is a clinical diagnosis and laboratory tests are not essential for the diagnosis. However, they are useful for excluding differential diagnoses in patients who do not exhibit the classic tetrad on presentation as well as for determining the prognosis. In scenarios where presentations are atypical and diagnoses are uncertain, tissue biopsy might be required to confirm the diagnosis. There are two main classification criteria for IgAV in children: the American College of Rheumatology (ACR) criteria (1990) and EULAR/PRINTO/PRES criteria (2010) [4,5]. Table 1 illustrates the respective criteria for both classifications. In adult cases, the EULAR/PRINTO/PRES criteria are more suitable due to the absence of validated diagnostic classification criteria for IgAV in adults. EULAR/PRINTO/PRES does not take into account age at onset and includes joint and renal involvement as well as IgA deposits as part of its diagnostic criteria, making it a better diagnostic tool with higher sensitivity than the ACR criteria.

**Table 1:** ACR 1990 criteria* vs. EULAR/PRINTO/PRES 2010 criteria** for IgAV *[4]; **[5] ACR: American College of Rheumatology; IgAV: immunoglobulin A vasculitis

Classification criteria	ACR 1990 [4]	EULAR/PRINTO/PRES 2010 [5]
Sensitivity	87.1%	100%
Specificity	87.7%	87%
Diagnostic criteria	At least 2 of the following criteria: age ≤20 years at disease onset, palpable purpura, diffuse abdominal pain, biopsy showing granulocytes in the walls of arterioles or venules	Purpura or petechiae and at least 1 of the following 4 criteria: abdominal pain, arthralgia or arthritis, renal involvement, leukocytoclastic vasculitis with predominant IgA deposits or proliferative glomerulonephritis with predominant IgA deposits

Elevated inflammatory markers such as white cell count, ESR, and CRP are not uncommon. The coagulation profile should be normal and platelet count is expected to be normal or elevated in order to support the diagnosis as thrombocytopenia would indicate a diagnosis other than IgAV. A low hemoglobin level should raise the suspicion of possible gastrointestinal bleeding. Serum complement levels are usually normal but can be low due to the activation of complement pathways by preceding infections. Serum IgA level can be elevated in up to 50% of cases but it is not considered a diagnostic marker and does not have a significant role in disease prognostication [3]. Renal function and urinalysis should be an essential part of the assessment to determine the presence of acute kidney injury, proteinuria, and hematuria.

Due to the low incidence of IgAV and more frequent atypical presentation of skin rashes in the adult population, biopsy plays an important role as a diagnostic confirmation tool. Light microscopy of skin lesion classically shows leukocytoclastic vasculitis prominently in postcapillary venules and direct immunofluorescence demonstrates IgA with C3 immune complex depositions in small vessels of the superficial dermis [6]. Although IgA immune complex deposits are notably associated with IgAV, it is not a pathognomonic finding for IgAV as it can be present in conditions like hypersensitivity vasculitis and cryoglobulinemia. Of note, IgA deposits have a sensitivity of 81% and specificity of 83% with a positive predictive value of 84% and a negative predictive value of 81% [7].

Currently, there are no definitive guidelines as to when to perform a renal biopsy for diagnosis confirmation. Renal biopsy is highly recommended when there is impaired kidney function, nephrotic or nephritic syndrome, persistent proteinuria >1 g/day at six months despite being on renin-angiotensin-aldosterone system inhibitors, or diagnostic uncertainty [3]. Although our patient did not have the aforementioned clinical presentation, renal biopsy was done due to initial diagnosis uncertainty at that point. Besides, the renal biopsy also plays a role in evaluating the extent of renal involvement. In IgAV nephritis (IgAVN), light microscopy findings include mesangial proliferation, mesangial hypercellularity, and crescent formation [8]. Immunofluorescence study demonstrates mesangial IgA1 and C3 immune complexes deposition. Histologically, IgAVN and IgA nephropathy are indistinguishable from each other. A proper clinical picture is essential to differentiate between these two conditions as IgAV is more of a systemic disease that affects multiple organ systems whereas IgA nephropathy primarily affects the kidneys. These two diseases are believed to share common pathogenesis and are often believed to be different manifestations of a single disease process. However, the clinical course is vastly different as IgAV is a one-hit disease whereas IgA nephropathy is a chronic progressive glomerular disease [9]. We arrived at our diagnosis based on several positive findings. Our patient presented with the classic tetrad, which is highly suggestive of IgA vasculitis. However, due to its rarity in adults, skin biopsy and renal biopsy were done, which eventually confirmed our diagnosis.

Most cases of IgAV are benign and self-limiting in children. On the contrary, the clinical course is more severe with a poorer prognosis in adults. Management of IgAV in adults is extremely challenging due to the lack of correlation between the initial presentation and long-term renal outcomes [10]. Patients who present initially with mild symptoms might progress to end-stage renal disease (ESRD) and, on the other hand, those who present with severe symptoms may achieve spontaneous remission. Up to 20% of adult patients experienced relapses, which is very uncommon in children [11]. A retrospective cohort study by Pillebout et al., which involved 250 adults with IgAV, reported that 13% of patients progressed to severe renal failure [estimated glomerular filtration rate (eGFR) <30 mL/minute] and 11% of patients ended up with ESRD [2]. Impaired baseline renal function, baseline proteinuria >1 g/day, macroscopic hematuria, hypertension, and persistent proteinuria >1 g/day during follow-up are all poor renal prognostic factors and associated with progression to ESRD. Renal biopsy histological findings associated with poor renal outcomes include interstitial fibrosis, glomerular sclerosis, and fibrinoid necrosis [2].

At present, there are no clear treatment guidelines for IgAV in adults. The majority of the recommendations were extrapolated from studies performed in pediatric populations. Mild disease with non-necrotic purpura, mild arthralgia, microscopic hematuria, mild proteinuria, and normal renal function can be managed with supportive measures that include rest, adequate hydration, and analgesia. NSAIDs should be avoided given the potential risk of renal and gastrointestinal involvement. Angiotensin-converting enzyme inhibitors are recommended in cases with mild-moderate proteinuria and hypertension.

Corticosteroids use remains controversial and debatable. Studies regarding corticosteroids use have only been done in the pediatric population. Three randomized placebo-controlled trials have been carried out in the pediatric age group. Ronkainen et al. included 171 children in their study to evaluate the efficacy of early prednisone therapy in preventing renal symptoms and treating renal and extrarenal symptoms. Prednisone (1 mg/kg/day for two weeks with weaning over the subsequent two weeks) was effective in alleviating abdominal pain and joint pain but did not prevent the development of renal symptoms [12]. Huber et al. included 40 children in their study to assess whether early corticosteroid use reduces the rate of renal or gastrointestinal complications. They reported that early prednisone (2 mg/kg/day for a week with weaning over the second week) did not reduce the risk of renal or gastrointestinal complications [13]. Lastly, Jauhola et al. reported that although prednisone alleviates symptoms related to IgAV, it did not alter the clinical course in 223 children [14]. There are several limitations to these studies: diagnosis of IgAV was not biopsy-proven; the short duration of corticosteroid therapy; and follow-up duration was less than a year. Besides, there is no available data from randomized controlled trials comparing the use of glucocorticoid versus supportive treatment for IgAV with renal involvement in adults. In fact, current recommendations by Kidney Disease: Improving Global Outcomes (KDIGO) guidelines are based on studies performed in patients with IgA nephropathy. One landmark randomized controlled trial by Locatelli et al. included patients with IgA nephropathy with proteinuria of 1-3.5 g/day and serum creatinine <1.5 mg/dL. These patients received intravenous methylprednisolone 1 g/day for three consecutive days at the beginning of months one, three, and five, and oral prednisone 0.5 mg/kg on alternate days for six months [15]. The study showed that those patients who received steroids had a significantly better 10-year renal outcome compared to the patients who received only supportive measures [15]. The corticosteroids regime has since been named as Pozzi-Locatelli regimen and it has been the recommended treatment by KDIGO for patients with IgAVN with moderate/severe proteinuria and eGFR >50 mL/minute [16].

Cyclophosphamide use is controversial as a randomized trial by Pillebout et al. has demonstrated that cyclophosphamide did not show significant benefit compared to treatment with steroids alone [17]. Studies of rituximab use have been limited to several case series, and successful remissions have been reported in cases refractory to corticosteroids therapy [18]. A retrospective study by Ren et al. reported that patients treated with mycophenolate mofetil (MMF) achieved a higher disease remission rate, suggesting that a combination of MMF with low-dose steroids may be as effective as high-dose steroids alone [19]. Data with regard to cyclosporine use is relatively scarce. Case series by Kalliakmani et al. have described five patients who achieved complete or partial remission at the five-year follow-up after treatment with a combination of cyclosporine and prednisone [20]. There are no available studies about azathioprine use in adults with IgAV. However, studies in pediatric populations have shown promising results as azathioprine was shown to improve the clinical course of nephritis as well as induce disease remission; however, these results involved small sample sizes.

## Conclusions

In summary, adult-onset IgAV is a rare disease associated with severe clinical course and poor outcomes. Due to the high frequency of renal involvement and associated poor prognosis, physicians should consider IgAV as a differential diagnosis when patients present with the classic tetrad. Management of adult-onset IgAV with severe renal involvement is undoubtedly not as straightforward compared to its pediatric counterpart due to the lack of available clinical trials in adult populations. Large randomized controlled trials are required in adult populations in order to guide better management in the future.
